# Development of a risk classification model in early pregnancy to screen for suboptimal postnatal mother-to-infant bonding: A prospective cohort study

**DOI:** 10.1371/journal.pone.0241574

**Published:** 2020-11-04

**Authors:** Elke Tichelman, Jens Henrichs, François G. Schellevis, Marjolein Y. Berger, Huibert Burger

**Affiliations:** 1 Department of Midwifery Science, Amsterdam UMC, AVAG, Amsterdam Public Health Research Institute, Vrije Universiteit Amsterdam, Amsterdam, The Netherlands; 2 Department of General Practice & Elderly Care Medicine, University Medical Centre Groningen, University of Groningen, Groningen, The Netherlands; 3 Department of General Practice & Elderly Care Medicine, Amsterdam UMC, Amsterdam Public Health Research Institute, Vrije Universiteit Amsterdam, Amsterdam, The Netherlands; 4 NIVEL, Netherlands Institute for Health Services Research, Utrecht, The Netherlands; 5 Department of Psychiatry, Amsterdam University Medical Center, Academic Medical Center, University of Amsterdam, Amsterdam, The Netherlands; University of Mississippi Medical Center, UNITED STATES

## Abstract

**Background:**

Previous studies identified demographic, reproduction-related and psychosocial correlates of suboptimal mother-to-infant bonding. Their joint informative value was still unknown. This study aimed to develop a multivariable model to screen early in pregnancy for suboptimal postnatal mother-to-infant bonding and to transform it into a risk classification model.

**Methods:**

Prospective cohort study conducted at 116 midwifery centers between 2010–2014. 634 women reported on the Mother-to-Infant Bonding questionnaire in 2015–2016. A broad range of determinants before 13 weeks of gestation were considered. Missing data were described, analyzed and imputed by multiple imputation. Multivariable logistic regression with backward elimination was used to develop a screening model. The explained variance, the Area Under the Curve of the final model were calculated and a Hosmer and Lemeshow test performed. Finally, we designed a risk classification model.

**Results:**

The prevalence of suboptimal mother-to-infant bonding was 11%. The estimated probability of suboptimal mother-to-infant can be calculated: *P(MIBS≥*4*) =* 1/(1+exp(-(-4.391+(parity× 0.519)+(Adult attachment avoidance score× 0.040))). The explained variance was 14% and the Area Under the Curve was 0.750 (95%CI 0.690–0.809). The Hosmer and Lemeshow test had a p-value of 0.21. This resulted in a risk classification model.

**Conclusion:**

Parity and adult attachment avoidance were the strongest independent determinants. Higher parity and higher levels of adult attachment avoidance are associated with an increased risk of suboptimal mother-to-infant bonding. The model and risk classification model should be externally validated and optimized before use in daily practice. Future research should include an external validation study, a study into the additional value of non-included determinants and finally a study on the impact and feasibility of the screening model.

## Introduction

Mother-to-infant bonding is defined as the emotional tie experienced by a mother towards her (unborn) child having the aim to protect the child [1]. The unidirectional mother-to-infant bond is not to be confused with the bidirectional infant-to-mother attachment based on which the infant uses the caregiver as secure base for exploration [2]. The development of mother-to-infant bonding already starts during pregnancy and remains stable until toddlerhood [1, 3] and has important implications. According to a recent systematic review, mother-to-infant bonding quality measured at any time point during pregnancy or postpartum is positively associated with mother-to-infant bonding quality at follow-up, either prenatally or postpartum [4].

Mother-to-infant bonding is essential for positive socio-emotional development of the child [5–7], and predicts child executive functioning [6]. Several explanations for these associations have been identified. Suboptimal mother-to-infant bonding is associated with more parenting stress and less sensitive and poorer maternal parenting styles and skills [8]. Less sensitive and poorer maternal parenting, in turn, has been linked to insecure attachment, depression, and anxiety in children [8]. Furthermore, women with suboptimal mother-to-infant bonding show less interest in their child’s health, and engage more frequently in negative health behaviours during pregnancy [9]. These behaviours are associated with adverse birth outcomes and with adverse long-term cognitive and socio-emotional development of the child [9].

Suboptimal mother-to-infant bonding is not uncommon. The prevalence of suboptimal mother-to-infant bonding one year postpartum varies between 5% and 11% in the general population indicating its potential to have a substantial impact on public health [10, 11]. Nevertheless, monitoring mother-to-infant bonding is presently no standard practice in pregnancy [12]. Although there are already adequate instruments to assess prenatal mother-to-infant bonding, they have not been validated before mid-pregnancy [13]. Before twenty weeks of gestation accurate assessment of mother-to-infant bonding is not possible. To target monitoring strategies and early interventions efficiently, a risk classification model will help to identify women at an early stage at risk of suboptimal postnatal mother-to-infant bonding. To our knowledge, no screening model for the general population exists. One investigation tried to establish prenatal determinants of mother-to-infant bonding in a clinical sample of 251 women enrolled at a public perinatal psychiatric service [14]. Previous studies identified demographic, reproduction-related and psychosocial correlates for impaired bonding in the general population [4]. However, their joint informative value is still unknown.

This is unfortunate as timely recognition of high risk of suboptimal bonding is an opportunity for health care professionals to provide preventive interventions at an early stage, i.e. from the beginning of pregnancy onwards. For example, midwives and gynecologists may stimulate mother-to-infant bonding by several interventions which are currently available, such as antenatal group education with attention to bonding and fetal movements, and interaction with the fetus by touching or singing [15–17]. Healthcare providers could also spend more time to address transition to motherhood early in pregnancy, for example by stimulating to fantasize about the baby during ultrasound assessment [12].

The overall aim of the current study is therefore to develop a multivariable model to screen early in pregnancy for mother-to-infant bonding postpartum. We investigated which determinants in the first trimester of pregnancy have the highest informative value for suboptimal mother-to-infant bonding up to 24 months postpartum and how to combine them into a reliable and easy to use risk classification model. Candidate determinants were derived a priori from reviews focusing on correlates of prenatal mother-to-infant bonding [18–20], and from one review of correlates of prenatal and postnatal mother-to-infant bonding [4].

## Methods

### Study design and setting

We used data from the Pregnancy, Anxiety and Depression (PAD) Study [21]. This prospective population-based cohort study investigates symptoms of and risk factors for anxiety and depression of the mother during pregnancy and the first years postpartum.

All pregnant women in their first trimester of pregnancy visiting a total of 109 primary and nine secondary obstetric care centers in the Netherlands between 2010 and 2014 were invited to participate. Ethical Approval for the study was obtained in 2010 and additional in 2015 from the medical ethical review board of the University Medical Center Groningen (METc2009.235). Written informed consent was obtained from all women. Privacy was guaranteed in accordance with Dutch legislation.

Only a single inclusion criterion was applied, i.e. the ability to read and speak Dutch.

Because of logistic factors, we were unable to determine the number of women that had been initially invited and out of these how many participated. Therefore, no initial response rate could be calculated. In total 4157 women gave informed consent. They filled in some of the baseline assessments [21]. Follow-up measures on mother-to-infant bonding used for the present study were collected in 2015 and 2016. Hence, the eligible population for the present analysis consisted of all women with a child aged between six and 24 months (n = 1,275). Women with multiple gestation (n = 12) were excluded from the current analyses. To enhance the quality of this study, and to enhance the transparency of our approach the TRIPOD statement for reporting the development of a multivariable prediction model for Individual Prediction or Diagnosis was followed [22].

### Measurements

#### Outcome variable

To assess postpartum mother-to-infant bonding the Dutch version of Mother-to-Infant Bonding Scale (MIBS) was used [23]. This self-report questionnaire consists of 8 items (loving, resentful, neutral or felt nothing, joyful, dislike, protective, disappointed and aggressive) using a 4-point Likert scale ranging from “Very much” to “Not at all”. Scores on the MIBS range between 0 and 24, with high scores indicating a poor mother-to-infant bond. The MIBS is used between six and 24 months postpartum [10, 23]. The sample of women varied in terms of the postpartum month assessment time point. Therefore, we investigated with a logistic regression analyses if scores on the MIBS were stable over time. Some studies used a score of 2 or more on the MIBS to indicate poor bonding [10, 11, 24]. However, none of these studies addressed whether the cut-off of 2 was the most optimal cut-off. Neither did these studies address the effect of changing the cut-off value on test characteristics such as sensitivity and specificity or predictive values. The MIBS was the best available measure since Matsunaga et al. investigated in the most optimal cut-off score of the MIBS. A score of 4 or more on the MIBS was used to define suboptimal bonding. Matsunaga et al. investigated the most optimal cut-off score of the MIBS among 723 mothers. The optimal cut off scores were 4/5 after 1 month postpartum [25]. This was based on an optimal tradeoff between sensitivity and specificity. When it comes to defining the outcome variable for a screening model for mother-to-infant bonding based on a rating scale such as the MIBS, a sufficiently high specificity of the MIBS itself is in our view more important than a high sensitivity. This is because a high specificity is associated with relatively few false positives. Too many false positive test may lead to an overestimation of suboptimal mother-to-infant bonding, and thereby evoking unnecessary worry among mothers. Some items of the MIBS address maternal feelings towards the baby which may directly lead to child abuse, a woman who indicated to have such feelings was also captured by a score of 4 or more on the MIBS in this study. The internal consistency of the Dutch MIBS in our study was comparable to previous studies which showed acceptable reliability and validity [21].

#### Candidate determinants

We applied four criteria for candidate determinants from four systematic reviews [4, 18–20]: plausibility (prior probability of being informative), reliability of assessment, and the determinant distribution (i.e. prevalence sufficiently high). Furthermore, we aimed at a maximum number of determinants of 10% of the number of women with suboptimal bonding [22].

Based on these criteria we selected prenatal depressive symptoms, prenatal anxiety, social support, adult attachment anxiety, adult attachment avoidance, domestic violence in history, marital status, feelings about pregnancy, maternal age and parity [4, 18–20]. Except for adult attachment style all candidate determinants were measured before 13 weeks of pregnancy.

Prenatal depressive symptomatology was measured and analyzed continuously with the Dutch version of the Edinburgh Postnatal Depression Scale (EPDS) [26]. Scores on this 10-item, 4-point Likert scale range from 0 to 30. Higher scores indicate more prenatal depressive symptomatology. A cut off score of ≥11 indicates a risk of minor or major depression during the first trimester of pregnancy^.^ The EPDS has been validated for use among pregnant women [26]. The Dutch version of the EPDS has shown good internal validity with a Cronbach's alpha of 0.83 [26].

Prenatal anxiety was measured with the Dutch version of the 6-item State and Trait Anxiety Inventory (STAI) on a 4-point Likert scale and was analyzed as a continuous variable. Scores range from 20 to 80 and a score of 42 or higher indicates clinically significant anxiety [27]. The 6-item STAI has similar reliability as the original 20-item STAI and has been validated to use among pregnant women [27]. The Cronbach's alpha coefficient for the 6-item STAI was 0.82 [27].

Social support was measured and analyzed continuously with the satisfaction score of the abbreviated version of the Social Support Questionnaire (SSQ). Scores on this 6-item, 6-point Likert scale range from 6 to 36. Higher scores indicate more satisfaction with social support [28]. The satisfaction score measures the individual's degree of satisfaction with the perceived support available, and has shown good reliability and validity [28]. The Cronbach's alpha coefficient for the SSQ Satisfaction subscale was 0.94 [28].

Adult attachment was measured with the Experiences in Close Relationships questionnaire (ECR) [29]. Adult attachment style was measured during the same postpartum period as the MIBS. The ECR is a 36-item self-report measure of adult attachment containing two 18-item subscales assessing dimensions of adult attachment, Anxiety and Avoidance. These two subscales were analyzed continuously. The Anxiety subscale taps fears of abandonment and rejection. The Avoidance subscale assesses discomfort with dependence and intimate self-disclosure. Each item is rated on a 7-point scale where 1 = strongly disagree and 7 = strongly agree [29]. Higher scores indicate higher attachment related anxiety or avoidance. Adult attachment style was measured during the same postpartum period as the MIBS under the assumption that subscales anxiety and avoidance remain stable over time as well as over a two month period as across a life span [30]. We have also tested this assumption in data from the PROMISES trial [31]. The results of the test-retest reliability of the two subscales of the ECR at 13 weeks of pregnancy and at twelve months postpartum indicated consistent scores over time. The intraclass correlation coefficient for the subscale anxiety was r = 0.75 (95% CI 0.61–0.84) and for the subscale avoidance r = 0.85 (95% CI 0.78–0.90) (S1 and S2 Tables). Cronbach’s alpha for adult attachment anxiety is 0.91 and for avoidance 0.94 [29].

A history of domestic violence was measured and analyzed dichotomously and defined as being mentally or physically abused by your partner or having a conflict with your partner.

Marital status and positive feelings about pregnancy were likewise measured and analyzed dichotomously as married/not married as well as positive feeling and no positive feelings about pregnancy at approximately 13 weeks of pregnancy.

Data on maternal age and parity were extracted from medical records. The persons who extracted these data were blinded for the mother-to-infant bonding scores.

#### Maternal characteristics

Maternal characteristics were measured at approximately 13 weeks of gestation and included: educational attainment level (elementary and lower tract of secondary education, higher tract of secondary education, higher vocational and university education) and ethnic background. Prematurity (yes/no) and intended breastfeeding (yes/no)were extracted from medical records. The age of the infant at the time mother-to-infant bonding was measured simultaneously with the MIBS.

### Data analysis

We calculated descriptive statistics for the outcome variable, the maternal characteristics and the candidate determinants. We compared characteristics of the included women and the non-responders on the MIBS and calculated the fraction of missing data for each candidate determinant of the responders of the MIBS. We studied the missing data mechanism by predicting missingness (yes/no) of data for each of these variables from the other variables using multivariable logistic regression analyses [32]. The final imputation model included all variables used in the analyses and all variables that predicted missingness of a certain variable, or its value. Under the assumption that data was missing at random (MAR) or completely at random [32], twenty datasets were imputed by chained equations and pooled according to Rubin’s rules [32]. Because MIBS scores were available for only 50% of the participants, we refrained from their imputation. We repeated our analyses using a complete case approach as a sensitivity analysis.

We present univariable odds ratios for suboptimal postnatal mother-to-infant bonding per determinant. Subsequent logistical regression analyses included all determinant variables irrespective of their univariable association as it has been shown that univariable preselection can lead to unstable models [33]. The following steps of model development were followed in each of the imputed data sets: (1) logistic regression with backward elimination of determinants with the Akaike Information Criterion (AIC) stopping rule [21]. This rule corresponds to a P-value of 0.157 for a determinant with one degree of freedom. The final model was defined as the set of determinants which were selected in at least 50% of all imputed datasets. (2) estimation of regression coefficients in the final model using logistic regression and (3) estimation of a shrinkage factor in each imputed data set. The averaged shrinkage factor over the imputed data sets was applied to the coefficients of the final model as a way of internal validation, i.e. to correct for overfitting. Shrinkage factors were estimated by bootstrapping using 250 bootstrap samples.

The following pooled performance measures of the final model were calculated: explained variance (Nagelkerke’s R2), the Hosmer and Lemeshow test, and the Area Under the Curve (AUC).

We checked if the results of our final analysis changed by adding the age of the child at assessment in the prediction model. We used Swets's criteria to assess the AUC [34]. The AUC of 0.5 to 0.6 is defined as bad performance, 0.6 to 0.7 as poor performance, 0.7 to 0.8 as satisfactory performance, 0.8 to 0.9 as good performance, and 0.9 to 1.0 as excellent performance [34].

Based on the regression coefficients of the final pooled screening model we created a risk classification model, which can be used to classify women at low, intermediate or high-risk for suboptimal mother-to-infant bonding. Low risk is defined as a estimated probability for suboptimal mother-to-infant bonding below 25%, intermediated risk between 25 and 75% and high risk as above 75%. Except for multivariable determinant selection, the level of significance was set at 0.05, two sided.

Statistical analyses were performed with SPSS Statistics 25.0 (SPSS inc. Chicago, Illinois) and with ‘RStudio’ statistical software (Version 1.1.463, R Development Core Team).

## Results

**Fig 1:**
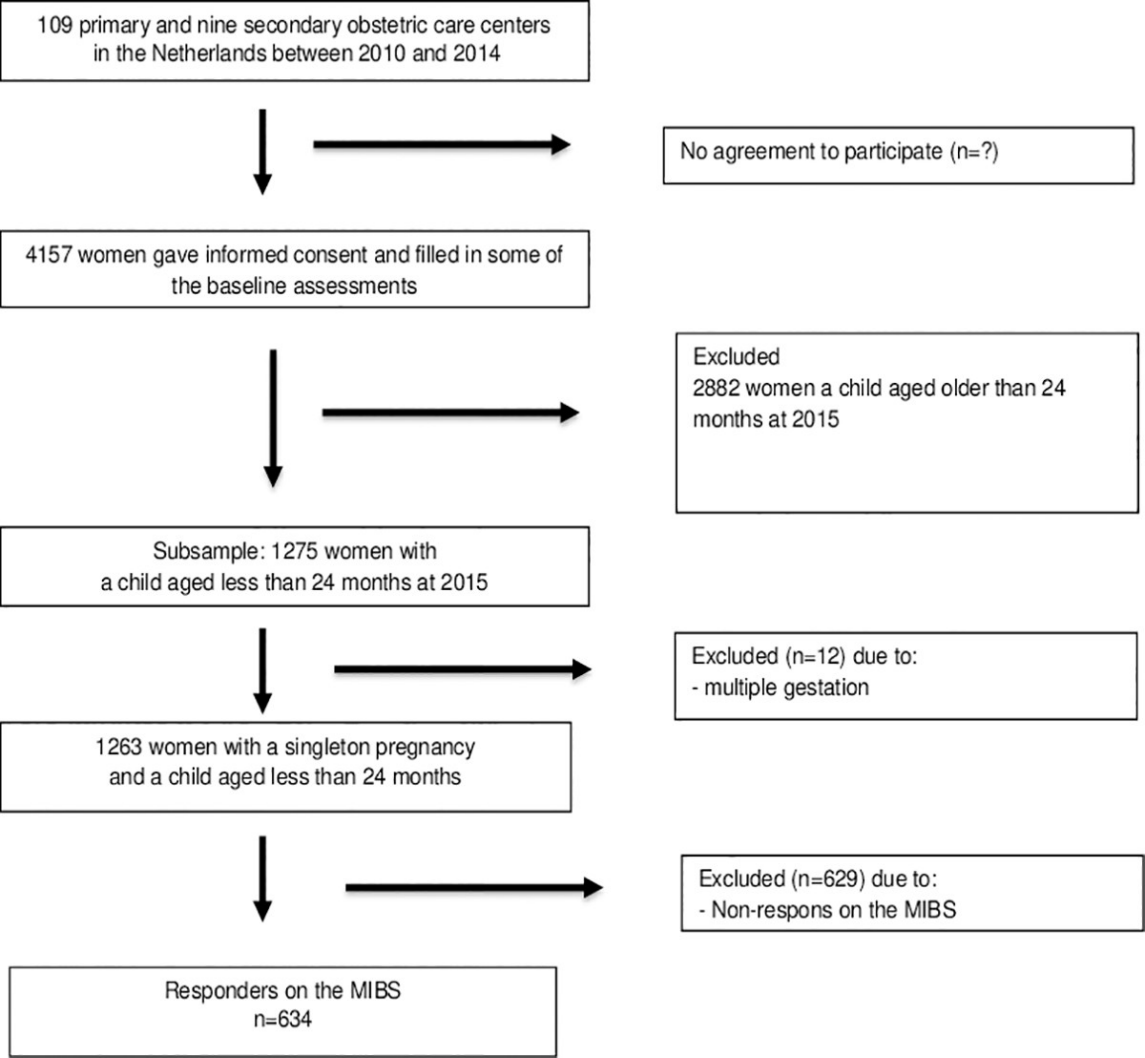
Flowchart.

The eligible population for the present analysis consisted of all women after singleton pregnancy with a child aged less than 24 months (n = 1,263). Of these, 634 responded by filling out the questionnaires, yielding a response rate of 50.2%. Fig 1 illustrates the flowchart.

Non-responders did not substantially nor statistically significantly differ from responders on depressive symptoms, anxiety, social support, maternal age, marital status, ethnicity, breastfeeding and Apgar score after 5 minutes. However, non-responders were significantly more often multiparous women (66% versus 54%, p<0.05) (Table 1). Reasons for not responding were mainly because respondents had not actively communicated changes to their home address or email address.

**Table 1:** Characteristics of the responders and the non-responders on the MIBS and the fraction of missing data of the responders.

	% missing data	Responders on MIBS N = 634	Non responders on MIBS n = 629
Characteristics first trimester of pregnancy			
Educational attainment level, n (%)	21%		
elementary and lower tract of secondary education		16 (3%)	18 (5%)
higher tract of secondary education		138 (28%)	106 (31%)
higher vocational or university education		344 (69%)	216 (64%)
Ethnic background, non-Dutch, n (%)	21%	20 (4%)	21 (6%)
Candidate determinants			
Maternal age, mean (sd)	3%	33.5 (4.5)	32.8 (4.8)
Marital status, married, n (%)	21%	228 (46%)	155 (46%)
Domestic violence in history, n (%)	26%	53 (11%)	28 (9%)
Parity* multiparous, n (%)	32%	232 (54%)	248 (66%)
No positive feelings about pregnancy, n (%)	33%	95 (22%)	54 (19%)
Prenatal depressive symptoms (EPDS), mean (sd)	30%	4.7 (3.7)	4.7 (3.8)
Prenatal anxiety (STAI), mean (sd)	29%	32.9 (8.5)	32.5 (9.2)
Social Support satisfaction score(SSQ), mean (sd)	32%	31.8 (4)	31.4 (5.4)
Adult attachment Avoidance (ECR), mean (sd)	25%	40.9 (16.3)	NA
Adult attachment Anxiety (ECR), mean (sd)	26%	48.8 (16.4)	NA
Characteristics at birth			
Preterm birth**	0%	35 (6%)	16 (3%)
Breastfeeding ***	2%	531 (85%)	99 (73%)
Characteristics 6 to 24 months postpartum			
Infants age in days, mean (sd) range	0%	530 (133) 183–730	536 (134) 183–730

EPDS = Edinburgh Postnatal Depression Scale, MIBS = Mother-to-Infant Bonding Scale, STAI = State Trait Anxiety Inventory, SSQ = Social Support Questionnaire, ECR = Experiences in Close Relationships questionnaire, NA = not applicable

* non-parametric: Mann-Whitney U test and Chi square test p = 0.001

** Chi square test p = 0.007

*** Chi square test p = 0.001

Of the 634 included women, 339 women (53%) had a score on the Mother-to-Infant Bonding Scale less than 2, 150 women (24%) had a score of 2, 77 women (12%) had a score of 3, 36 women (6%) had a score of 4 and 32 women (5%) had a score of ≥5. The proportion of missing data of candidate determinants varied between 3% and 33%. Maternal age varied from 22 to 45 years. The results of a logistic regression analysis showed that the MIBS-scores were not significant associated with the age of the child (days postpartum) (p-value of 0.897).

Table 2 presents the univariable associations of all candidate determinants with suboptimal mother-to-infant bonding. Out of ten, six candidate determinants were significantly associated with a higher risk of suboptimal mother-to-infant bonding: adult attachment avoidance, adult attachment anxiety, prenatal depressive symptoms (measured continuously), prenatal anxiety symptoms (measured continuously), no positive feelings about pregnancy and parity. Women with a suboptimal MIBS score were more often multiparous than nulliparous women, respectively 44 (65%) versus 287 (51%). Women with a suboptimal MIBS score were not significantly different in prenatal anxiety and depression measured dichotomously (Table 2).

**Table 2:** Univariable associations in the imputed datasets between candidate determinants and suboptimal mother-to-infant bonding (MIBS ≥4).

Candidate determinants	MIBS≥4	MIBS<4	Univariable OR	P value
	n = 68 (%)	n = 566 (%)	MIB≥4 (95% CI)	
Maternal age			1.049 (0.991–1.110)	0.100
Marital status, married	35 (51%)	311 (55%)	0.880 (0.489–1.583)	0.668
Domestic violence in history	15 (22%)	70 (12%)	1.964 (0.964–3,927)	0.063
Parity			1.544 (1.098–2.172)	0.013
No positive feelings about pregnancy	24 (35%)	126 (22%)	1.954 (1.063–3.589)	0.031
Prenatal depressive symptoms			1.106 (1.031–1.186)	0.005
Prenatal depression (EPDS) ≥11	9 (13%)	42 (7%)	1.843 (0.728–4.663)	0.196
Prenatal anxiety symptoms			1.037 (1.004–1.071)	0.030
Prenatal anxiety STAI ≥42, n (%)	10 (15%)	61 (11%)	1.380 (0.583–3.267)	0.462
Quality Social Support SSQ			0.939 (0.879–1.003)	0.060
Adult attachment ECR avoidance			1.040 (1.022–1.058)	<0.001
Adult attachment ECR anxiety			1.032 (1.014–1.050)	0.001

EPDS = Edinburgh Postnatal Depression Scale, MIBS = Mother-to-Infant Bonding Scale, STAI = State Trait Anxiety Inventory, SSQ = Social Support Questionnaire, ECR = Experiences in Close Relationships questionnaire, OR = Odds Ratio

In the first step of the model development, parity and adult attachment avoidance were included in all datasets. The variable prenatal depressive symptoms was included in six of the 20 datasets. Adult attachment anxiety, social support and no positive feelings about pregnancy were included in three of the 20 datasets. Marital status and prenatal anxiety symptoms were included in two datasets.

Therefore, we included parity and adult attachment avoidance in the final model. We estimated the pooled multivariable regression coefficients in all datasets. The pooled shrinkage factor was 0.99. Table 3 describes the final model. The final formula for calculating the estimated probability of suboptimal mother-to-infant is: *P(MIBS≥*4*) =* 1/(1+exp(–(-4.3914 + (parity × 0.518698) + (Adult attachment avoidance × 0.040430863))).

**Table 3:** The full model to allow estimations for individuals (including all regression coefficients and model intercept).

Intercept and determinants	β ^a^
Intercept ^b^	-4.3914
Parity	0.518698
Adult attachment avoidance	0.040430863
**Performance measures of the full model**^**b**^	
Nagelkerke’s R^2^	0.138
Hosmer-Lemeshow goodness of fit test (p-value)	0.210
ROC area (95% CI)	0.750 (0.690–0.809)

ROC = receiver-operating characteristic.

* The estimated probability of suboptimal mother-to-infant bonding can be calculated using the following formula: *P(MIBS≥*4*) =* 1/(1+exp(–(-4.3914 + (parity × 0.518698) + (Adult attachment avoidance × 0.040430863)).

^a^ Regression coefficient multiplied with a shrinkage factor (obtained from the bootstrapping procedure) of the intern validation procedure

^b^ pooled measures (from the imputed datasets)

We checked if the results of our final analysis changed by adding the age of the child at assessment in the model. However, the age of the child did not contribute to the model.

The pooled explained variance, determined with Nagelkerke’s R2 was 14%. The Hosmer and Lemeshow test had a p-value of 0.21 which indicates a good fit. Finally, pooled AUC was 0.750 with a 95% CI of 0.690–0.809, which is classified as satisfactory performance [30].

**Fig 2:**
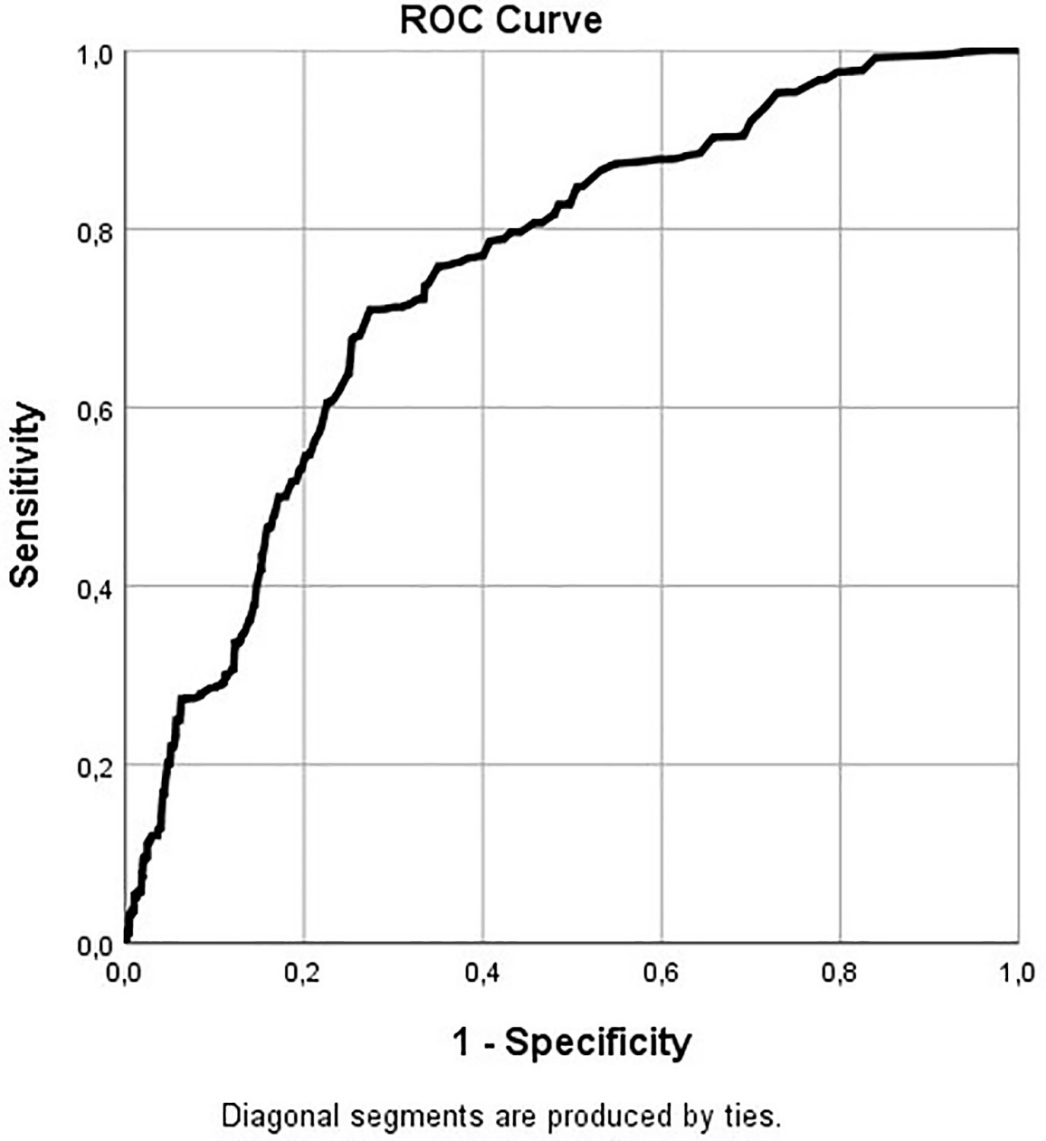
The pooled receiver-operating characteristic of the full model.

Fig 2 presents the pooled receiver-operating characteristic curve of the final model. Table 4 presents a practical risk classification model, based on the regression coefficients of the final model, which can be used to classify women as low, intermediate or high-risk for suboptimal mother-to-infant bonding based on information on parity and scores on the Experiences in Close Relationships questionnaire which measures (among others) adult attachment avoidance. The complete case analysis showed similar results.

**Table 4:** Risk classification model for suboptimal mother-to-infant bonding, based on the regression coefficients of the final model.

	Risk of suboptimal mother-to-infant bonding		
	Low	Intermediate	High
Determinants			
Parity	Adult attachment avoidance, scores based on the ECR		
0	<82	82–126	–
1	<69	69–122	123–126
2	<56	56–110	111–126
3	<43	43–97	98–126
4	<30	31–84	85–126

ECR = Experiences in Close Relationships questionnaire

Low risk is defined as an estimated probability for suboptimal mother-to-infant

Bonding (*MIBS≥*4) below 25%, intermediated risk between 25 and 75% and high risk as above 75%.

## Discussion

In this study we developed a multivariable model to screen early in pregnancy for mother-to-infant bonding postpartum. Parity and adult attachment avoidance were the strongest independent determinants of suboptimal postnatal mother-to-infant bonding and formed the final model.

Higher parity and higher levels of adult attachment avoidance were associated with an increased risk of suboptimal mother-to-infant bonding. The model showed satisfactory performance. Based on the results, we designed an easy to use risk classification model for detection of suboptimal bonding. This risk classification model enables health care professionals to easily classify women at risk for suboptimal mother-to-infant bonding.

Parity and adult attachment avoidance were the strongest independent determinants of suboptimal postnatal mother-to-infant bonding. The informative value of parity can be explained as follows. When a woman becomes a mother for the first time, she may have more time and attention for her pregnancy and child which may positively affect mother-to-infant bonding [31]. Some mothers having their second, third or fourth child feel concerned about bonding. They worry about whether they will have just as much love for their new arrival as they do for their older child [35]. The findings of this study were mentioned before in other studies. Parity was mentioned as a determinant in a systematic review, although mostly in combination with prenatal mother-to-infant bonding. In 7 of 17 included studies of this review it was reported, that women expecting a second or third child had lower mother-to-infant bonding. Reported correlations were mostly weak [4].

The finding that higher adult attachment avoidance is associated with an increased risk of suboptimal mother-to-infant bonding is in line with the findings of a recent cross sectional study [36]. Anxious adult attachment style was not included in our final model. Apparently, avoidant adult attachment style was a stronger determinant than anxious adult attachment style. An explanation for why adult attachment avoidance is a determinant of suboptimal mother-to-infant bonding could be that adult attachment avoidance or insecurity is considered a mediator, or partial mediator of the association between experienced childhood maltreatment and depression, anxiety or behavioural problems in the next generation [37–39]. Therefore, it seems relevant to implement the identification of maternal attachment style of pregnant women into prevention or intervention strategies designed to reduce children's risk for behavioural problems. Such strategies should be combined with attachment-based interventions that are widely available and have been demonstrated to modify parenting behaviours [40].

In this study depressive symptoms were not included in the final model. This is in contrast with other literature which showed that postnatal depressive symptoms were consistently correlated with postnatal mother-to-infant bonding [4]. An explanation may be that adult attachment is related to postnatal depressive symptoms [41], and possibly overruled depressive symptoms in our analysis. During pregnancy adult attachment can act as a vulnerability factor for the development of depressive symptoms in the subsequent postnatal period [41]. Apparently, adult attachment appeared in our analysis to be the stronger independent determinant. It should be borne in mind here that the determinants in our risk model are not necessarily causal factors for suboptimal bonding. This is because the data analysis aimed to identify variables that jointly had high informative performance rather than to unravel the factors involved in the aetiology of bonding problems [42].

The explained variance of the screening model of suboptimal bonding in the general population of the model was relatively low (14%). However, it must be emphasized that the explained variance only concerns one aspect of performance, i.e. the overall quality of a screening model performance in a given data set [43, 44], and therefore interpretation in absolute terms must be cautious. The other two aspects of model performance are central in evaluating the performance and they are calibration and discrimination [45]. In the current study, the Hosmer and Lemeshow test had a p-value of 0.21 which indicates a good model calibration and fit. Finally, the pooled AUC was 0.750 with a 95% CI of 0.690–0.809, which is classified as satisfactory discrimination performance [34]. Mother-to-infant bonding is a complex construct determined by multiple determinants. We cannot completely rule out, that additional determinants may be involved which were not assessed in our study. Nevertheless, this study adds important knowledge to the complex construct of mother-to-infant bonding. Only a small number of candidate determinants surfaced as independent determinants. A possible explanation can be the limited statistical power associated with the relatively low number of women with suboptimal bonding in our sample. That is, 68 mothers had suboptimal bonding with an MIBS score ≥4.

Contrary to our results, Farré-Sender et al. found that emotional abuse in childhood, family psychiatric history, previous psychiatric hospitalization, and anxiety during pregnancy were the main determinants of mother-to-infant bonding. However, this was investigated in a clinical sample of 251 women enrolled at a public perinatal psychiatric service. The results showed a model explaining 10.7% of the variance. Aspects like calibration and discrimination were not reported in this study [14].

### Strengths and limitations

To our knowledge, this is the first study to develop a multivariable model to screen early in pregnancy for suboptimal postnatal mother-to-infant bonding in the general population. Strengths of this study were the inclusion of a broad range of determinants all selected for analyses based on literature and the design of the study. All the three aspects of model performance, including calibration and discrimination were investigated [45]. The model showed satisfactory performance. Another strength of this study is the heterogeneity of the population caused by unselective population-based inclusion and a mixed recruitment setting of both primary and nine secondary care centers and the which likely contributes to greater generalizability [46]. The sample from primary midwifery care and secondary obstetrics care was likely to be unselected with respect to mother-to-infant bonding and the determinants.

Some limitations should be addressed regarding the interpretation of our results. First, the outcome variable was available in only 50% of the eligible population. Therefore, we decided not to impute mother-to-infant bonding scores. This rate of attrition could suggest selection bias and could decrease the generalizability of the results. Furthermore, this non-response may have resulted in bias within the determinants. Non-responders on the MIBS were more often multiparous women which may have caused an underestimation of the prevalence of suboptimal mother-to-infant bonding and of the strengths of the observed associations. However, this is unlikely to have caused spurious associations. Secondly, adult attachment was measured cross-sectionally with the outcome. Before this study, we tested the assumption that adult attachment remains stable from pregnancy to motherhood in the PROMISES trial [31]. The intraclass correlation coefficient of the subscales anxiety and avoidance are indicating respectively moderate and good test re-test reliability [47], indicating consistency over time. Our assumption that adult attachment remains stable over time has also been supported by previous literature [30]. On the other hand, the pregnancy and birth of a child can be transforming for women in many ways. Pregnancy offers a window of opportunity for future health of both the mother and the child. On average women engage more frequently in positive health behaviours during pregnancy [9]. These behaviours are associated with good birth outcomes and healthier mothers [9]. However, based on the existing literature it is still debatable whether in mothers with insecure attachment the birth of the child could transform in terms of adult attachment representations. Several studies in the adult attachment literature have demonstrated continuity of attachment organization in general [48–50]. Furthermore, as far as we know, adult attachment measured by the Experiences in Close Relationships questionnaire has only once been investigated in a prospective cohort study following pregnant women [30]. The results of our study and the study of Kooiman both showed that subscales anxiety and avoidance remain stable over time. Finally, Stern et al. examined change in first-time mothers’ adult attachment style across the first 2 years of motherhood [51]. 162 economically stressed primiparous mothers completed measures of adult attachment anxiety and avoidance at five time points: when their children were 0, 6, 12, 18, and 24 months of age. Converging results of stability functions and latent growth curve models suggest that attachment styles were generally stable during the first 2 years of motherhood, even in this economically stressed sample [51]. Our results add to the growing insight in adult attachment development over time and suggest a long-term stability.

Although adult attachment and mother-to-infant bonding can be seen as conceptually-close measures, they measure different constructs [1]. Mother-to-infant bonding measured the feelings and emotions a mother experiences towards her child. On the other hand the adult attachment questionnaire we used, measured adult attachment towards the partner. The anxiety subscale taps fears of abandonment and rejection, especially towards the partner. The avoidance subscale assesses discomfort with dependence and intimate self-disclosure towards the partner. Recently, other authors describe the constructs as different constructs as well [4, 52]. We cannot completely rule out that there is the slight possibility that the way the mother responded to one questionnaire affected the way she responded to the second one.

Finally, this study investigated the development and internal validation of a screening model. Yet, externally validating the final model in another population remains essential. A third limitation is that the PAD-Study is a screening study, in which there was no immediate request for help from the respondents at inclusion.

### Clinical and scientific implications

Prenatal care visits by midwives and gynecologists take place from early in pregnancy onwards. However, so far mother-to-infant bonding is neglected during such visits, as only in two percent of such visits mother-to-infant bonding was addressed by primary care midwives in the Netherlands [12]. Our risk classification model enables health care professionals to identify in pregnancy women at risk for suboptimal mother-to-infant bonding. For this, they can take into account the pregnant women’s parity and adult attachment style. The model showed satisfactory performance. Therefore, we recommend to invest in an external validation and updating of the model. We also recommend to identify more possible determinants of suboptimal mother-to-infant bonding using large-scale longitudinal research. The screening model may serve as a starting point for further research and as a starting point for developing supporting material in clinical practice. Our model may also have added value to clinical practice. Whether the informative accuracy of this model in all women is adequate is also a matter of judgment and depends on available alternatives for risk stratification [46]. Currently, our model is the only screening model available for mother-to-infant bonding in the general population and may be used with the understanding that it should serve as a clinical guide rather than a prescriptive tool. Nevertheless, the impact of this model for patient relevant outcomes is not yet known. In the future, and after external validation, an impact study should, idealiter quantify the effect of using this screening model on the behaviour of health care professionals, outcomes, and costs effectiveness of care using the model compared with usual care [47]. The risk classification model can be useful to plan monitoring strategies, interventions and prevention, in order to support the development of both motherhood and parenting skills. Health care professionals can thereby help to individualize patient care plans. We advise investigating the capacity of health care professionals to systematically screen for adult attachment style in pregnancy.

Adult attachment style in this study was tapped by the 36-item questionnaire [29]. The disadvantage of this questionnaire is the length of this questionnaire. For practical use, we recommend to investigate in a shorter questionnaire measuring both adult attachment avoidance and adult attachment anxiety.

## Conclusion

Higher parity and higher levels of adult attachment avoidance are independently associated with an increased risk of suboptimal mother-to-infant bonding. The developed screening model showed satisfactory performance measures. Based on the results, we designed a practical risk classification model for early detection of suboptimal bonding. The screening model should be externally validated and optimized before use in daily practice. The model may serve as a starting point for further research. Future research should include an external validation study, a study into the additional value of non-included determinants and finally a study on the impact and feasibility (such as the capacity of health care professionals to screen for adult attachment in pregnancy) of the screening model.

## Supporting information

S1 Table Results of paired sampled t-tested of the two subscales of the ECR in the PROMISES trial. (DOCX)

S2 Table Results of the test-retest reliability of the two subscales of the ECR in the PROMISES trial. (DOCX)

S3 Table TRIPOD Checklist: Prediction model development and validation. (DOCX)

S1 File Patient data information. (SAV)
