# Supplementary material for: Development of a risk classification model in early pregnancy to screen for suboptimal postnatal mother-to-infant bonding: A prospective cohort study
Source: PLoS One. 2020 Nov 4;15(11):e0241574. doi: 10.1371/journal.pone.0241574 (PMC7641412; doi:10.1371/journal.pone.0241574)
Supplement: S1 Table — (DOCX) [file pone.0241574.s001.docx]

**S1 Table.** Results of paired sampled t-tested of the two subscales of the ECR in the PROMISES trial.

|  | 13 weeks of gestation  Mean (sd) | 12 months postpartum  Mean (sd) | Mean difference  (95% confidence interval) | P value |
| --- | --- | --- | --- | --- |
| Adult attachment ECR avoidance  Adult attachment ECR anxiety | 44.41  62.08 | 45.78  61.46 | -1.37 (-4.05-1.31)  0.62 (-2.97-4.20) | 0.313  0.732 |

We have tested the assumption that adult attachment remains stable from pregnancy to motherhood in the PROMISES trial (Meijer et al., 2011). The results of the test-retest reliability of the two subscales of the ECR at 13 weeks of pregnancy and at twelve months postpartum indicated consistent measurements over time. The paired sampled t-tested showed no significant differences at the two subscales (See table S1).

Reference

Meijer, J. L., Bockting, C. L., Beijers, C., Verbeek, T., Stant, A. D., Ormel, J., Stolk, R. P., de Jonge, P., van Pampus, M. G., … Burger, H. (2011). PRegnancy Outcomes after a Maternity Intervention for Stressful EmotionS (PROMISES): study protocol for a randomised controlled trial. Trials, 12, 157. doi:10.1186/1745-6215-12-157
